# Tracking Consensus for Nonlinear Multi-Agent Systems Under Asynchronous Switching and Undirected Topology

**DOI:** 10.3390/s25154760

**Published:** 2025-08-01

**Authors:** Shanyan Hu, Mengling Wang

**Affiliations:** 1 Key Laboratory of Smart Manufacturing in Energy Chemical Process, Ministry of Education, East China University of Science and Technology, Shanghai 200237, China; 14789159961@163.com; 2 State Key Laboratory of Fluid Power and Mechatronic Systems, Zhejiang University, Hangzhou 310027, China

**Keywords:** tracking consensus, asynchronous switching, dynamic event-triggered mechanism, multi-agent systems

## Abstract

This paper investigates the tracking consensus of nonlinear multi-agent systems under undirected topology, considering asynchronous switching caused by delays between communication topology switching and controller switching. First, by using the properties of undirected topology graphs, the controller design process is simplified. Then, to address asynchronous delays during topology switching, the system operation is divided into synchronized and delayed modes based on the status of the controller and topology. Every operating mode has a corresponding control strategy. To alleviate the burden of communication and computation, an event-triggered mechanism (ETM) is introduced to reduce the number of controller updates. By constructing an augmented Lyapunov function that incorporates both matching and mismatching periods, sufficient conditions ensuring system stability are established. The required controller based on the dynamic ETM is obtained by solving Linear Matrix Inequalities (LMIs). Finally, a simulation example is conducted to verify its effectiveness.

## 1. Introduction

Multi-agent systems (MASs) can carry out coordinated tasks in industrial manufacturing through cooperative control, boosting production efficiency. MASs have been widely applied in various fields, including robotic systems [1], unmanned surface vehicles [2], and wireless sensor networks [3]. As an important topic in cooperative control, the tracking consensus has attracted sustained attention [4], and the related literature continues to emerge in the expansion of complex scenarios. The research scope covers practical constraints, such as communication delays [5] and packet losses [6]. In such systems, the design of control protocols remains the primary challenges of MASs. Traditional control protocols assume that agents have access to continuous and uninterrupted state information. However, the communication bandwidth between agents is often restricted, which poses significant challenges to the design of control protocols. To address this issue, researchers have proposed control protocols based on an event-triggered mechanism (ETM) [7]. This mechanism drives control updates and exchanges information based on specific events, which effectively reduces unnecessary sampling and control updates, thus improving resource efficiency. Due to these advantages, the ETM has attracted extensive research for various types of MASs [8,9,10,11,12].

For most studies addressing consensus, agents usually adopt a fixed communication topology to exchange information. However, in nonlinear multi-agent cooperative control [13,14], when systems execute collaborative tasks in real-world environments, the communication topology often experiences frequent and unpredictable changes. These fluctuations may result from dynamic link conditions due to obstacle avoidance, communication interference, or link failures. Because of these dynamics, classic control systems that rely on fixed topologies become ineffective. Therefore, it is crucial to incorporate the switching topologies into control design and analysis. The main focus of research is designing control protocols that guarantee consensus and system stability under time-varying communication structures.

At present, extensive research has been devoted to the analysis of MASs under switching topologies [15,16,17,18,19]. Most of these studies adopt topology-independent controller designs, in which a single controller is applied on different switching topologies. To enhance adaptability, topology-dependent control strategies are introduced, allowing specific controllers to be designed for different topologies. Xia and Yu [20] investigated the tracking consensus problem and extended the controller to switching topologies in simulations. Reference [21] studied the fixed-time tracking consensus for second-order MASs. Reference [22] researched the tracking consensus for switching topologies under a dynamic event-triggered mechanism (DETM). Reference [23] addressed the distributed adaptive control problem for connected vehicles with stochastic switching topologies. The aforementioned studies on switching topologies primarily focus on synchronous switching, where the controller responds instantly to changes in communication topology. However, in real industrial processes, delays caused by triggering devices and network latency may lead to a mismatch between the controller and the communication topology. This phenomenon is called asynchronous switching [24]. To solve the time delay caused by the asynchronous switching of the controller and the communication topology in practical applications, this work has developed a controller for tracking control in asynchronous switching scenarios.

In [16,25], the issue of asynchronous consensus was investigated for the case of multiple topological modes with detection delays. In these studies, a distributed asynchronous switching observer was developed for followers to estimate the leader’s state. However, the observer-based control protocol requires constant measurement sampling and persistent neighbor communication, which leads to high energy consumption and frequent control updates. Reference [26] designed an asynchronous event-triggered controller based on system matrix modes to resolve the mismatch between controller and system modes. While effective for single-agent systems, this method is only applicable to such systems and has not been extended to MASs. Reference [27] addressed asynchronous cooperative output regulation under connected switching topologies by relating maximum detection delays to minimum match-interval dwell times, designing a heterogeneous adaptive controller using distributed asynchronous observer states. A key aspect not addressed in this work is explicit energy consumption optimization, crucial for practical scenarios. This paper fully considers the time delay between the switching of the communication topology and the controller. The system operation is divided into synchronized and delayed periods between the communication topology and the controller. A DETM and a controller design method are proposed for asynchronous switching.

Considering the issues mentioned above, this work develops a dynamic event-triggered control law, aiming to achieve the tracking consistency of nonlinear MASs under asynchronous switching. The main contributions are fourfold.

(1) According to the characteristics of the undirected topology graph, the asynchronous switching tracking consensus problem is transformed into the asynchronous switching leaderless consensus. Specifically, by using the bidirectional connectivity of undirected graphs, the state error between the leader and the followers is defined, and the tracking objective of the leader is reformulated as an internal error convergence problem among the followers using a weighted Laplacian matrix *H*.

(2) To address the asynchronous switching delay issue, a topology-dependent time-delay feedback control protocol is proposed. Based on the topology activation status and the modes of a multi-modal controller, synchronized and delayed controllers are designed using weighted Laplacian matrices. The asynchronous switching process is divided into a multi-modal synchronous process consisting of synchronized and delayed periods.

(3) For asynchronous switching, we propose a distributed dynamic event-triggered strategy based on undirected topology. The mechanism uses a threshold condition that combines the measurement state ${\hat{q}}_{i}\left(t\right)$ and measurement errors to determine controller update instants. Since ${\hat{q}}_{i}\left(t\right)$ directly links to the communication topology’s weighted Laplacian matrix $H_{\sigma }$, this design autonomously adjusts update timing while responding instantly to topological changes. This approach allows multiple followers to accurately track the leader’s trajectory while reducing communication resource consumption.

(4) We design a public form Lyapunov function integrating asynchronous switching properties. This function remains non-increasing at the moment of topology switching to ensure persistent system stability. Meanwhile, we construct an augmented Lyapunov function embedding an internal dynamic variable to eliminate the Zeno behavior. Through the regulation capability of the variable, a strict positive lower bound is guaranteed for the event trigger interval, thereby ensuring that the switching behavior is achievable in the timing sequence.

## 2. Preliminaries

This section introduces the graph theory and switched systems.

### 2.1. Graph Theory

Let G={V,E,A} denote a communication topology graph consisting of *N* agents. $V=\{v_{1},v_{2},\ldots ,v_{N}\}$ represents the set of nodes and E⊆(V×V) denotes the set of edges. $A=\left[a_{ij}\right]\in R^{N\times N}$ is the adjacency matrix. For an undirected graph G, $a_{ij}=a_{ji}$ for any nodes $v_{j}$ and $v_{i}$ always holds. The Laplacian matrix of graph G is $L=\left[l_{ij}\right]\in R^{N\times N}$ with elements,

$$l_{ij}=\left\{\begin{array}{cc} -a_{ij} & i=j\\ \sum_{j=1,j\neq i}^{N}a_{ij} & i\neq j \end{array}\right.$$

The degree matrix is $D=diag\{d_{1},d_{2},\ldots ,d_{N}\}$, and the relationship among matrices L,D,A is given by L=D−A. In a communication topology graph with a leader, the matrix $M=\mathrm{diag}\{m_{1},m_{2},\ldots ,m_{N}\}$ represents the communication relationship between the leader and followers. When $m_{i}>0$, it indicates that follower *i* receives information from the leader. The weighted Laplacian matrix is expressed as H=L+M.

### 2.2. Switching System

A switched system is defined by several subsystems and a switching signal σ(t). The system’s dynamic behavior is governed by different subsystems during different time intervals, and the switching signal governs the active subsystem at time *t*. Essentially, the σ(t) is a time-based function that indicates the active subsystem at each point in time. Specifically, by considering multiple communication topologies as different subsystems, the system switches among these topologies under the control of the switching signal, thereby forming a dynamic system model with switching topology characteristics.

To characterize the switching mechanism, this paper introduces a piecewise constant function σ(t):[0,∞)→S={1,…,p,q,…m}, where *S* represents the set of all possible communication topologies. When σ(t)=i, the current topology of the system corresponds to the communication graph *i*. In this way, the switching process among multiple network structures is flexibly described.

## 3. Problem Formulation

This work considers MASs consisting of a leader and *N* followers. The dynamics of the follower is described by the following equation:

(1) $$\begin{array}{c} {\dot{x}}_{i}\left(t\right)=Ax_{i}\left(t\right)+Bf(x_{i}\left(t\right),t)+Bu_{i}\left(t\right) \end{array}.$$

$x_{i}\left(t\right)\in R^{n}$ denotes the *n*-dimensional state vector of agent *i*; $u_{i}\left(t\right)\in R^{p}$ is the *p* dimensional control input; and $f(x_{i}\left(t\right),t)$ represents a nonlinear function, where i=1,2,…,N. The matrices A and B are system matrices. The leader’s dynamics is

(2) $$\begin{array}{c} {\dot{x}}_{0}\left(t\right)=Ax_{0}\left(t\right)+Bf\left(x_{0}\left(t\right),t\right). \end{array}$$

The leader’s state is denoted by $x_{0}\left(t\right)\in R^{n}$. The leader broadcasts information to followers via unidirectional links, with no reverse communication. Its state evolution is unaffected by follower states.

Before introducing the controller, the following basic assumptions need to be introduced for further analysis.

**Assumption** **1.** *(A,B) in* (1) *and* (2) *is stabilizable.*

**Assumption** **2.** *For the nonlinear function f(·) in* (1) *and* (2)*, it holds that for any $x_{i}\left(t\right)$,$x_{j}\left(t\right)\in R^{n}$,$∥f\left(x_{i}\left(t\right),t\right)-f\left(x_{j}\left(t\right),t\right)∥⩽d∥x_{i}\left(t\right)-x_{j}\left(t\right)∥$, where d≥0.*

**Assumption** **3.** *$G_{\sigma (t)},\forall \sigma \left(t\right)=p\in S$ is fixed and connected during the time interval $\left[t_{s},t_{s+1}\right)$.*

**Assumption** **4.** *Boundedness on switching delay $\tau (t_{s})$: the detection delay signal $\tau (t_{s})$ is constrained by $\tau \left(t_{s}\right)<t_{s+1}-t_{s}$ in the interval $\left[t_{s},t_{s+1}\right)$.*

**Lemma** **1** ([28])**.** *Let $Q\in R^{n\times n}$ be a positive-definite matrix and $P\in R^{n\times n}$ be symmetric. Then, the following inequality is satisfied.*

$$x^{T}Px\leq \lambda_{max}\left(Q^{-1}P\right)x^{T}Qx.$$

For the analysis of switched systems in this paper, we adopt the average dwell time (ADT) method. The definition of the ADT is provided below.

**Definition** **1.** *Denote by $N_{\sigma }\left(t_{1},t_{2}\right)$ the number of switching events between communication topologies within the interval $\left(t_{1},t_{2}\right)$. The following inequality is satisfied for every $t_{2}>t_{1}\geq 0$.*

$$N_{\sigma }\left(t_{1},t_{2}\right)⩽N_{0}+\frac{t_{2}-t_{1}}{h},$$

*where $N_{0}>0$ represents the chattering bound, and h>0 denotes the dwell time of switching signal σ(t).*

The mathematical definition of the tracking consensus for MASs is provided below.

**Definition** **2.** *Tracking consensus between systems* (1) *and* (2) *is achieved, provided that the following equation holds for any given initial condition.*

$$\underset{t\to \infty }{lim}∥x_{i}\left(t\right)-x_{0}\left(t\right)∥=0,\forall i=1,\ldots ,N$$

The objective of this research is to develop a distributed dynamic event-triggered strategy based on undirected topologies under asynchronous switching such that the nonlinear leader–follower MASs (1) and (2) achieve consensus. This strategy ensures that followers gradually track the leader from any initial state while reducing resource consumption.

## 4. Main Results

To achieve tracking consensus under asynchronous switching, this section proposes a dynamic event-triggered controller based on undirected topology. Furthermore, it is rigorously proven that Zeno behavior is excluded under the proposed control scheme.

### 4.1. Dynamic Event-Triggered Control Protocol Under Asynchronous Switching

We define a combined measurement variable,

(3) $$\begin{array}{c} q_{i}\left(t\right)=\sum_{j=1}^{N}a_{ij}^{\sigma (t)}(x_{j}\left(t\right)-x_{i}\left(t\right)+m_{i\sigma }\left(x_{0}\left(t\right)-x_{i}\left(t\right)\right),\forall i\in V \end{array}$$

Let ${\hat{x}}_{i}\left(t\right)=x_{i}\left(t_{k}^{i}\right)$, and then the state measurement error of the system is $e_{i}\left(t\right)={\hat{x}}_{i}\left(t\right)-x_{i}\left(t\right),t\in \left[t_{k}^{i},t_{k+1}^{i}\right)$, where $t_{k}^{i}$ is the triggering time of follower *i*. The tracking error is $\varepsilon_{i}\left(t\right)=x_{i}\left(t\right)-x_{0}\left(t\right)$, representing the error between the leader and follower. Based on the tracking error and (3), we have

(4) $$\begin{array}{c} q_{i}\left(t\right)=-\left(\sum_{j=1}^{N}\varepsilon_{j}\left(t\right)+m_{i\sigma }\varepsilon_{i}\left(t\right)\right). \end{array}$$

Define $q\left(t\right)={\left[q_{1}^{T}\left(t\right),q_{2}^{T}\left(t\right),\ldots ,q_{N}^{T}\left(t\right)\right]}^{T}$ and $\varepsilon \left(t\right)={\left[\varepsilon_{1}^{T}\left(t\right),\varepsilon_{2}^{T}\left(t\right),\ldots ,\varepsilon_{N}^{T}\left(t\right)\right]}^{T}$, and then (4) becomes $q\left(t\right)=-\left(H_{\sigma }⊗I_{N}\right)\varepsilon \left(t\right)$.

In the triggering interval $t\in [t_{k}^{i},t_{k}^{i+1})$, the combined measurement variable remains constant, $q_{i}\left(t\right)=q_{i}\left(t_{k}^{i}\right)$. Substituting this into (3), we obtain $q_{i}\left(t_{k}^{i}\right)=\sum_{j=1}^{N}a_{ij}^{\sigma (t)}({\hat{x}}_{j}\left(t\right)-{\hat{x}}_{i}\left(t\right)+m_{i\sigma }\left(x_{0}\left(t\right)-{\hat{x}}_{i}\left(t\right)\right),t\in \left[t_{k}^{i},t_{k+1}^{i}\right)$. Using the relationships between state information, $e_{i}\left(t\right)$, and $\varepsilon_{i}\left(t\right)$, we have

(5) $$\begin{array}{c} {\hat{q}}_{i}\left(t\right)=q_{i}\left(t_{k}^{i}\right)=-(\sum_{j=1}^{N}l_{ij}^{\sigma (t)}\left(\varepsilon_{j}\left(t\right)+e_{j}\left(t\right)\right)+m_{i\sigma }\left(\varepsilon_{i}\left(t\right)+e_{j}\left(t\right)\right). \end{array}$$

Meanwhile, denote $\hat{q}\left(t\right)={\left[{\hat{q}}_{1}^{T}\left(t\right),{\hat{q}}_{2}^{T}\left(t\right),\ldots ,{\hat{q}}_{N}^{T}\left(t\right)\right]}^{T}$, and the Kronecker form of $\hat{q}\left(t\right)$ is $\hat{q}\left(t\right)=-\left(H_{\sigma }⊗I_{N}\right)\varepsilon \left(t\right)-\left(H_{\sigma }⊗I_{N}\right)e\left(t\right)$.

The distributed control protocol for agent *i* is designed as

(6) $$\begin{array}{c} u_{i}=K_{\sigma (t-\tau )}q_{i}\left(t_{k}^{i}\right),t\in \left[t_{k}^{i},t_{k}^{i+1}\right) \end{array}.$$

where $K_{\sigma (t-\tau )},\forall \sigma \left(t-\tau \right)\in S$ is the feedback gain. σ(t−τ) denotes the controller switching signal, and τ is a delay that varies over time. Based on $\hat{q}\left(t\right)$, the structure of the closed-loop controller is expressed as $u\left(t\right)=-\left(H_{\sigma }⊗K_{\sigma (t-\tau )}\right)\varepsilon \left(t\right)-\left(H_{\sigma }⊗K_{\sigma (t-\tau )}\right)e\left(t\right)$.

**Figure 1:**
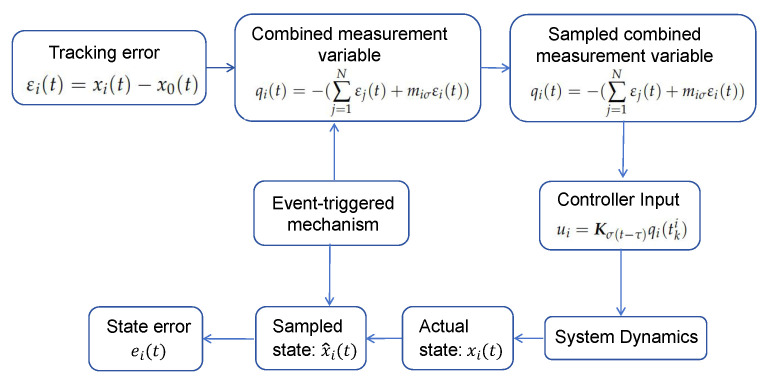
Schematic diagram of the relationship between the variables $q\left(t\right),\hat{q}\left(t\right)$, and e(t).

The relationship between the variables $q\left(t\right),\hat{q}\left(t\right)$, and e(t) is shown in Figure 1.

**Remark** **1.** *The control protocol* (6) *designs specific feedback gains for each graph $G_{\sigma }$, which are topology-dependent. Due to transmission delays or the time required to detect topology switching, a time delay $\tau (t_{s})$ is introduced in the control protocol. This delay affects the real-time responsiveness of the control protocol and constitutes a non-negligible factor in the system design.*

The dynamic equation of $\varepsilon_{i}\left(t\right)$ is calculated as ${\dot{\varepsilon }}_{i}\left(t\right)=A\varepsilon_{i}\left(t\right)+Bu_{i}\left(t\right)+B\left(f\left(x_{i}\left(t\right)\right)-f\left(x_{0}\left(t\right)\right)\right)$. Substituting the closed-loop controller matrix into this equation yields

(7) $$\begin{array}{cc} \dot{\varepsilon }\left(t\right) & =\left(I⊗A\right)\varepsilon \left(t\right)+\left(I⊗B\right)u\left(t\right)+\left(I⊗B\right)(f\left(x\left(t\right)\right)-1_{N}⊗f\left(x_{0}\left(t\right)\right))\\ \; & =\left(I⊗A-H⊗BK_{\sigma (t-\tau )}\right)\varepsilon \left(t\right)-\left(H⊗BK_{\sigma (t-\tau (t))}\right)e\left(t\right)+\left(I⊗B\right)F\left(x\left(t\right)\right). \end{array}$$

where $F\left(x\left(t\right)\right)={\left[f\left(x_{1}\left(t\right)\right)-f\left(x_{0}\left(t\right)\right),\ldots ,f\left(x_{N}\left(t\right)\right)-f\left(x_{0}\left(t\right)\right)\right]}^{T}$.

According to the $\varepsilon_{i}\left(t\right)$, the tracking consensus defined in (2) is achieved only when $\varepsilon_{i}\left(t\right)=0$, that is, $x_{1}-x_{0}=\ldots =x_{N}-x_{0}=0$. This implies that the tracking consensus can be reduced to the asymptotic stability analysis of the closed-loop system (7), which ensures that $\underset{t\to \infty }{lim}∥\varepsilon_{i}\left(t\right)∥=0,\forall i=1,\ldots ,N$ holds over the network.

The set $\left\{t_{k},k\in N\right\}$ represents the sequence of triggering times in the ETM. For follower *i*, the DETM that determines its triggering instants is

(8) $$\begin{array}{c} t_{k+1}^{i}=inf\left\{t>t_{k}^{i}\left|\rho_{1}e_{i}^{T}\left(t\right)e_{i}\left(t\right)-\rho_{2}{\hat{q}}_{i}^{T}\left(t\right){\hat{q}}_{i}\left(t\right)-\pi_{i}\eta_{i}\left(t\right)⩾0\right.\right\}. \end{array}$$

The dynamic variable $\eta_{i}\left(t\right)$ satisfies the following rule:

(9) $$\begin{array}{c} {\dot{\eta }}_{i}\left(t\right)=-\rho_{3}\eta_{i}\left(t\right)+c\left(\rho_{2}{\hat{q}}_{i}^{T}\left(t\right){\hat{q}}_{i}\left(t\right)-\rho_{1}e_{i}^{T}\left(t\right)e_{i}\left(t\right)\right). \end{array}$$

Additionally, the parameters satisfy $c,\rho_{1},\rho_{2},\rho_{3},\pi_{i}>0$. Since the DETM is applied exclusively to the followers, the tracking error is $e_{i}\left(t\right)={\hat{\varepsilon }}_{i}\left(t\right)-\varepsilon_{0}\left(t\right)$.

**Remark** **2.** *The design principle of the DETM determines the next triggering instant based on both the measurement error and measurement state ${\hat{q}}_{i}\left(t\right)$. The measurement state ${\hat{q}}_{i}\left(t\right)$ is intrinsically connected to the weighted Laplacian matrix Hσ. This connection enables the DETM to immediately respond to communication topology changes during agent connectivity variations. Therefore, this capability ensures efficient and stable operation of MASs.*

**Remark** **3.** *The distinction between the SETM and DETM lies in the incorporation of dynamic variables. When the dynamic variable η(t)=0, the DETM in Equation* (8) *reduces to a SETM. Importantly, compared to the SETM, the DETM allows for longer inter-event intervals due to the additional term $\pi_{i}\eta_{i}\left(t\right)>0$, which greatly lowers the probability of Zeno behavior.*

**Figure 2:**
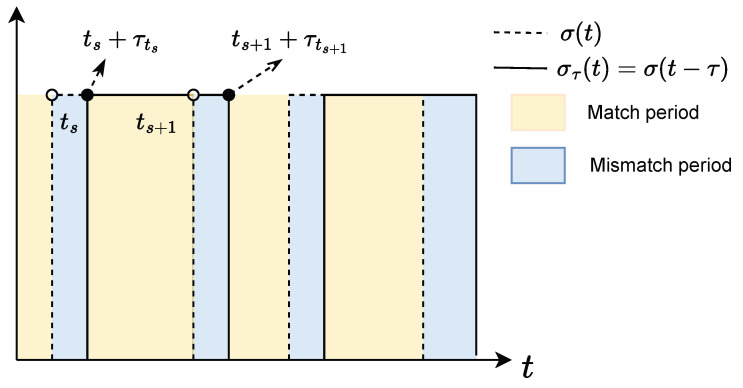
Asynchronous switching signal.

In this paper, we design a topology-dependent controller and investigate the asynchronous switching scenario where a time delay exists between communication topology switching and controller switching. Additionally, we emphasize the influence of this switching delay on the system performance and stability. As shown in Figure 2, the temporal evolution of the controller switching signal and the communication topology switching signal indicates the presence of a non-negligible time delay between them. This visualization provides an intuitive understanding of critical issues in asynchronous switching processes, providing a theoretical basis for the development and analysis of subsequent control strategies.

Let $\left\{t_{s},s\in N\right\}$ denote the switching instants of the signal σ(t) and $\tau (t_{s})$ denote the detection delay signal between the controller and the communication topology, which specifies the time delay over the interval $\left[t_{s},t_{s+1}\right)$. If the communication topology switching instants are $t_{s}$ and $t_{s+1}$, the corresponding controller switching instants become $t_{s}+\tau \left(t_{s}\right)$ and $t_{s+1}+\tau \left(t_{s+1}\right)$. The entire timeline is divided into two parts. The black part represents the mismatch periods between the controller and the communication topology, such as $\left[t_{s},t_{s}+\tau \left(t_{s}\right)\right)$. The yellow intervals are match periods, such as $\left[t_{s}+\tau \left(t_{s}\right),t_{s+1}\right)$.

**Remark** **4.** *Asynchronous switching causes a control mismatch within the interval $\left[t_{s},t_{s}+\tau_{s}\right)$ because of the delay $\tau_{s}$ between the topological structure switching and the controller switching. When $\tau_{s}$ is small enough, the delayed interval shortens and the controller switching instant $t_{s}+\tau_{s}$ comes closer to the topology switching instant $t_{s}$. Consequently, the system behavior is roughly similar to the synchronous switching mode, where the controller and topology transitions coincide nearly instantaneously.*

**Lemma** **2.** *The tracking consensus problem is addressed by the control law (6) when the following systems are asymptotically stable:*

(10) $$\begin{array}{cc} \; & {\dot{\tilde{\varepsilon }}}_{i}\left(t\right)=A{\tilde{\varepsilon }}_{i}\left(t\right)+B{\tilde{u}}_{i}\left(t\right)+B{\tilde{F}}_{i}\left(t\right),\\ \; & {\tilde{u}}_{i}\left(t\right)=-\lambda_{i\sigma }\left(H_{\sigma }\right)K_{\sigma (t-\tau (t))}\left({\tilde{\varepsilon }}_{i}\left(t\right)+{\tilde{e}}_{i}\left(t\right)\right). \end{array}$$

**Proof.**  For the weighted Laplacian matrix $H_{\sigma }$, there exists an orthogonal matrix $U_{p}$ for any σ(t)=p∈S, satisfying ${U_{p}}^{T}U_{p}=I$, such that the following equation holds:

(11) $$\begin{array}{c} U_{p}^{T}{\mathrm{HU}}_{p}=\Lambda =diag\left(\lambda_{1p}\left(H\right),\ldots ,\lambda_{np}\left(H\right)\right). \end{array}$$

Denote $\tilde{\varepsilon }\left(t\right)=\left(U_{p}^{T}⊗I_{N}\right)\varepsilon \left(t\right)={\left[{\tilde{\varepsilon }}_{1}^{T}\left(t\right),{\tilde{\varepsilon }}_{2}^{T}\left(t\right),\ldots ,{\tilde{\varepsilon }}_{N}^{T}\left(t\right)\right]}^{T}$, and $\tilde{e}\left(t\right)=\left(U_{\sigma }^{T}⊗I_{N}\right)e\left(t\right)={\left[{\tilde{e}}_{1}^{T}\left(t\right),{\tilde{e}}_{2}^{T}\left(t\right),\ldots ,{\tilde{e}}_{N}^{T}\left(t\right)\right]}^{T}$, and (7) is described as

(12) $$\begin{array}{cc} \; & \dot{\tilde{e}}\left(t\right)=\left(I_{N}⊗A\right)\tilde{\varepsilon }\left(t\right)+\left({U_{\sigma }}^{T}⊗B\right)u\left(t\right)+\left({U_{\sigma }}^{T}⊗B\right)F\left(t\right),\\ \; & u\left(t\right)=-\left(H_{\sigma }U_{\sigma }⊗K_{\sigma (t-\tau )}\right)\tilde{\varepsilon }\left(t\right)-\left(H_{\sigma }U_{\sigma }⊗K_{\sigma (t-\tau )}\right)\tilde{e}\left(t\right). \end{array}$$

Introduce the following transformation: $\tilde{q}\left(t\right)=\left(U^{T}⊗I_{N}\right)q\left(t\right)={\left[{\tilde{q}}_{1}^{T}\left(t\right),{\tilde{q}}_{2}^{T}\left(t\right),\ldots ,{\tilde{q}}_{N}^{T}\left(t\right)\right]}^{T}$, $\tilde{\hat{q}}\left(t\right)=\left(U^{T}⊗I_{N}\right)\hat{q}\left(t\right)={\left[{\tilde{\hat{q}}}_{1}^{T}\left(t\right),{\tilde{\hat{q}}}_{2}^{T}\left(t\right),\ldots ,{\tilde{\hat{q}}}_{N}^{T}\left(t\right)\right]}^{T}$, $\tilde{F}\left(x\left(t\right)\right)=\left(U^{T}⊗I_{N}\right)F\left(x\left(t\right)\right)={\left[{\tilde{F}}_{1}^{T}\left(t\right),{\tilde{F}}_{2}^{T}\left(t\right),\ldots ,{\tilde{F}}_{N}^{T}\left(t\right)\right]}^{T}$, and $\tilde{u}\left(t\right)=\left(U^{T}⊗I_{N}\right)u\left(t\right)={\left[{\tilde{u}}_{1}^{T}\left(t\right),\ldots ,{\tilde{u}}_{N}^{T}\left(t\right)\right]}^{T}$.

Further, convert (12) to

(13) $$\begin{array}{cc} \; & \dot{\tilde{\varepsilon }}\left(t\right)=\left(I_{N}⊗A\right)\tilde{\varepsilon }\left(t\right)+\left(I_{N}⊗B\right)\tilde{u}\left(t\right)+\left(I_{N}⊗B\right)\tilde{F}\left(x\left(t\right)\right),\\ \; & \tilde{u}\left(t\right)=-\left(\Lambda_{\sigma }⊗K_{\sigma (t-\tau (t))}\right)\left(\tilde{\varepsilon }\left(t\right)+\tilde{e}\left(t\right)\right). \end{array}$$

As (13) comprises *N* individual subsystems governed by (10), the asymptotic stability of each subsystem induces asymptotic stability in the system (7). □

**Remark** **5.** *By introducing the transformation matrix $U_{p}$ and modal coordinate transformation, the system analysis process is substantially simplified. By using the spectral decomposition of $H_{\sigma }$, this transformation ensures that the topological correlation characteristics in control input* (6) *are reduced to eigenvalue regulation through the diagonal matrix $\Lambda_{\sigma }$. This method avoids the computational complexity associated with a high-dimensional matrix. Consequently, it limits the asynchronous switching effects caused by time delays to local adjustments of the gain matrix $K_{\sigma (t-\tau )}$, significantly improving analytical efficiency.*

**Remark** **6.** *According to Lemma 2, the analysis of confirming the asymptotic stability of the system* (10) *can be used to solve the tracking consensus for systems* (1) *and* (2)*. The design of controllers under asynchronous switching is easier by the application of Lemma 2, which reduces the complexity of the controller design.*

According to Figure 2, consider the interval between successive topology switches $\left[t_{s},t_{s+1}\right)$. Within this interval, the communication topology remains fixed as $\sigma \left(t\right)=\sigma \left(t_{s}\right)$. The controller switching signal σ(t−τ) is affected by the delay τ, and its dynamic process is divided into two parts. During the delayed period $\left[t_{s},t_{s}+\tau \left(t_{s}\right)\right)$, σ(t−τ) remains consistent with the previous topology switching signal $\sigma (t_{s-1})$. In the synchronized period $\left[t_{s}+\tau \left(t_{s}\right),t_{s+1}\right)$, σ(t−τ) aligns with the current topology signal $\sigma (t_{s})$. The mathematical description is given by

$$\sigma \left(t-\tau \right)=\left\{\begin{array}{cc} \sigma (t_{s-1}), & t\in [t_{s},t_{s}+\tau \left(t_{s}\right))\\ \sigma (t_{s}), & t\in [t_{s}+\tau \left(t_{s}\right),t_{s+1}) \end{array}\right.$$

For any switching instances $\sigma \left(t_{s}\right)=p,\sigma \left(t_{s-1}\right)=q,\forall p,q\in S,p\neq q$, the control protocol in (10) is equivalent to

(14) $${\tilde{u}}_{i}\left(t\right)=\left\{\begin{array}{cc} -\lambda_{ip}K_{q}{\tilde{\varepsilon }}_{i}\left(t\right)-\lambda_{ip}K_{q}{\tilde{e}}_{i}\left(t\right), & t\in [t_{s},t_{s}+\tau \left(t_{s}\right))\\ -\lambda_{ip}K_{p}{\tilde{\varepsilon }}_{i}\left(t\right)-\lambda_{ip}K_{p}{\tilde{e}}_{i}\left(t\right), & t\in [t_{s}+\tau \left(t_{s}\right),t_{s+1}) \end{array}\right.$$

The transformed tracking error is equivalent to

$${\tilde{\varepsilon }}_{i}\left(t\right)=\left\{\begin{array}{cc} \left(A-\lambda_{ip}BK_{q}\right){\tilde{\varepsilon }}_{i}\left(t\right)+B\tilde{F}(x_{i}\left(t\right)-\lambda_{ip}BK_{q}{\tilde{e}}_{i}\left(t\right), & t\in [t_{s},t_{s}+\tau \left(t_{s}\right))\\ \left(A-\lambda_{ip}BK_{p}\right){\tilde{\varepsilon }}_{i}\left(t\right)+B\tilde{F}(x_{i}\left(t\right)-\lambda_{ip}BK_{p}{\tilde{e}}_{i}\left(t\right), & t\in [t_{s}+\tau \left(t_{s}\right),t_{s+1}) \end{array}\right.$$

Let ${\tilde{A}}_{ipq}=A-\lambda_{ip}BK_{q},{\tilde{A}}_{ipp}=A-\lambda_{ip}BK_{p},\forall p,q\in S$, then

(15) $${\tilde{\varepsilon }}_{i}\left(t\right)=\left\{\begin{array}{cc} {\tilde{A}}_{ipq}{\tilde{\varepsilon }}_{i}\left(t\right)+B\tilde{F}\left(x_{i}\left(t\right)-\lambda_{ip}BK_{q}{\tilde{e}}_{i}\left(t\right)\right), & t\in [t_{s},t_{s}+\tau \left(t_{s}\right))\\ {\tilde{A}}_{ipp}{\tilde{\varepsilon }}_{i}\left(t\right)+B\tilde{F}\left(x_{i}\left(t\right)-\lambda_{ip}BK_{p}{\tilde{e}}_{i}\left(t\right)\right), & t\in [t_{s}+\tau \left(t_{s}\right),t_{s+1}) \end{array}\right.$$

**Remark** **7.** *When $\sigma (t_{s})=p,\forall p\in S$, the topology $G_{p}$ is activated over the interval $t\in [t_{s},t_{s+1})$. However, due to the time delay $\tau (t_{s})$, this interval is divided into two distinct subintervals. During $t\in [t_{s}+\tau \left(t_{s}\right),t_{s+1})$, the controller remains synchronized with the topology and is thus referred to as the synchronized controller. In contrast, the controller is unable to synchronize with the current topological state within $t\in [t_{s},t_{s}+\tau \left(t_{s}\right))$ as it does not match the active topology. Throughout this subinterval, the subsystem is controlled by a delayed controller. This de-synchronization between the controller and the topology constitutes the asynchronous switching behavior exhibited by the system.*

**Figure 3:**
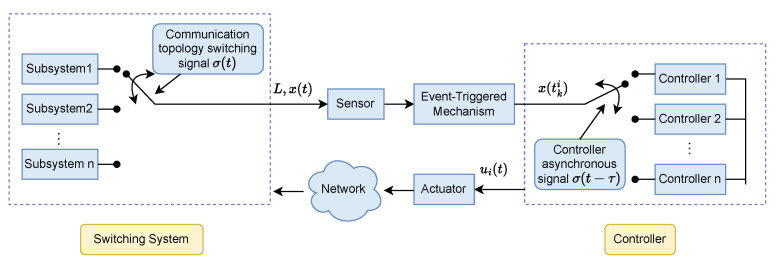
Control scheme of the agent *i*.

Figure 3 illustrates the control principle under asynchronous switching conditions. A distributed control protocol $u_{i}\left(t\right)$ and a DETM are designed for the *i*-th agent. To reflect the time lag, the switching of the topology structure is governed by the function σ(t), while the controller switching is regulated by the delayed switching signal σ(t−τ). The ETM determines whether the control input needs to be updated based on the agent’s current state $x_{i}\left(t\right)$ and specifies the next triggering instant $t_{k}^{i}$. Then, the controller computes the control input $u_{i}\left(t\right)$ using the information at this instant, achieving effective control under low-frequency triggering.

### 4.2. Consensus Analysis

**Lemma** **3.** *Let $\kappa_{1}$ and $\kappa_{2}$ be two classes of $\kappa_{\infty }$ functions. Suppose there exist functions $V_{\sigma (t)}\left(t\right),\sigma \left(t\right)\in S$ that make the inequalities* (16)–(18) *hold. Then, system* (10) *achieves global asymptotic consensus. Furthermore, the ADT of the switching signal satisfies* (19)*, where θ=α+β, and $T_{m}=max\tau \left(t_{s}\right)$.*

(16) $$\kappa_{1}\left(\left|\tilde{\varepsilon }\left(t\right)\right|\right)\leq V_{i}\left(\tilde{\varepsilon }\left(t\right)\right)\leq \kappa_{2}\left(\left|\tilde{\varepsilon }\left(t\right)\right|\right)$$

(17) $${\dot{V}}_{i}\leq \left\{\begin{array}{ccc} \beta V_{i}\leq 0, & \; & t\in [t_{s},t_{s}+\tau \left(t_{s}\right))\\ -\alpha V_{i}\leq 0, & \; & t\in [t_{s}+\tau \left(t_{s}\right),t_{s+1}) \end{array}\right.$$

(18) $$V_{i}\left(t_{s}\right)\leq \mu V_{i}\left(t_{s}^{-}\right),\forall i\in V$$

(19) $$h>\frac{\theta T_{m}+ln\mu }{\alpha }$$

**Proof** **of** **Lemma** **3.** Within the switching interval $t\in [t_{s},t_{s+1})$, the following formula is derived based on (17).

(20) $$\left\{\begin{array}{cc} V_{i}\left(t_{s}+\tau \left(t_{s}\right)\right)<e^{\beta \tau (t_{s})}V_{i}\left(t_{s}\right), & t\in [t_{s},t_{s}+\tau \left(t_{s}\right))\\ V_{i}\left(t\right)<e^{-\alpha (t-t_{s}-\tau \left(t_{s}\right))}V_{i}\left(t_{s}+\tau \left(t_{s}\right)\right)), & t\in [t_{s}+\tau \left(t_{s}\right),t_{s+1}) \end{array}\right.$$

Combining (18) with (20), we obtain

(21) $$\begin{array}{cc} V_{i}\left(t\right) & \leq e^{-\alpha \left(t-t_{s}\right)+\theta \tau \left(t_{s}\right)}V_{i}\left(t_{s}\right)\\ \; & \leq \mu e^{-\alpha \left(t-t_{s}\right)+\theta \tau \left(t_{s}\right)}V_{i}\left(t_{s}^{-}\right)\\ \; & \leq \ldots \\ \; & \leq \mu^{N_{\sigma (t)}\left(t_{0},t\right)}e^{-\alpha \left(t-t_{0}\right)+\theta \left(\tau \left(t_{s}\right)+\ldots +\tau \left(t_{1}\right)+\tau \left(t_{0}\right)\right)}V_{i}\left(t_{0}\right). \end{array}$$

Setting $t_{0}=0$, then $V_{i}\left(t\right)\leq \mu^{N_{0}\left(0,T\right)}e^{-\alpha t+\theta \left[N_{\sigma }\left(0,t\right)+1\right]T_{m}}V_{i}\left(0\right)$.

According to Definition 2 of the ADT, it has $N_{\sigma }\left(0,t\right)\leq N_{0}+\frac{t}{h}$, and the switching delay satisfies $\tau \left(t_{s}\right)\leq T_{m}$. It then follows that

(22) $$\begin{array}{cc} V_{i}\left(t\right) & \leq \mu^{N_{0}+\frac{t}{h}}e^{-\alpha t+\theta (T_{m}+N_{0}+\frac{t}{h})}V_{i}\left(0\right)\\ \; & \leq \mu^{N_{0}}e^{\theta (T_{m}+N_{0})}e^{(-\alpha +\frac{\ln\mu }{h}+\frac{\theta T_{m}}{h})t}V_{i}\left(0\right). \end{array}$$

Since $-\alpha +\frac{\ln\mu }{h}+\frac{\theta T_{m}}{h}<0$, $V_{\sigma (t)}\left(t\right)$ converges to 0 as t→∞. Furthermore, based on (16), it follows that the system achieves asymptotic stability. □

**Theorem** **1.** *For each agent i, assume there exists a positive-definite matrix $P_{ip}$ that satisfies the following inequality. In addition, if the parameters in the DETM* (8) *and* (9) *meet the specified inequalities and equations, then the nonlinear MASs described by* (10) *are guaranteed to be stable.*

$$\Pi_{i1}=\left[\begin{array}{cc} {\left({\tilde{A}}_{ipq}+d_{N}⊗B\right)}^{T}P_{ip}+P_{ip}\left({\tilde{A}}_{ipq}+d_{N}⊗B\right)-\beta P_{ip} & *\\ c\rho_{2}\lambda_{ip}^{2}I-\lambda_{ip}K_{q}^{T}B^{T}P_{ip} & c(\rho_{2}\lambda_{ip}^{2}-\rho_{1})I \end{array}\right]<0$$

$$\begin{array}{c} \Pi_{i2}=\left[\begin{array}{cc} {\left({\tilde{A}}_{ipp}+d_{N}⊗B\right)}^{T}P_{ip}+P_{ip}\left({\tilde{A}}_{ipp}+d_{N}⊗B\right)+\alpha P_{ip} & *\\ c\rho_{2}\lambda_{ip}^{2}I-\lambda_{ip}K_{p}^{T}B^{T}P_{ip} & c(\rho_{2}\lambda_{ip}^{2}-\rho_{1})I \end{array}\right]<0 \end{array}$$

$$P_{ip}<\mu P_{iq},c\rho_{2}\lambda_{ip}^{2}I-\lambda_{ip}K_{q}^{T}B^{T}P_{ip}>0$$

$$\pi_{i}>0,c>0,\rho_{2}\lambda_{ip}^{2}-\rho_{1}<0$$

*where $\rho_{3}>\alpha >0,\beta >0$, and μ>1.*

**Proof** **of** **Theorem** **1.** A model-dependent Lyapunov function for each agent *i* in the system (13) is designed:

$$V_{1}\left(t\right)={\tilde{\varepsilon }}^{T}\left(t\right)\left(I_{N}⊗P_{\sigma }\right)\tilde{\varepsilon }\left(t\right),V_{2}\left(t\right)=\sum_{i=1}^{N}\eta_{i}\left(t\right).$$

The topology-based DETM defined in (8) ensures that the inequality (23) is always valid.

(23) $$\rho_{1}e_{i}^{T}\left(t\right)e_{i}\left(t\right)-\rho_{2}{\hat{q}}_{i}^{T}\left(t\right){\hat{q}}_{i}\left(t\right)-\pi_{i}\eta_{i}\left(t\right)\leq 0.$$

When the internal dynamic variable (9) is introduced,

$${\dot{\eta }}_{i}\left(t\right)\geq -\rho_{3}\eta_{i}\left(t\right)-c\pi_{i}\eta_{i}\left(t\right),$$

(24) $$\eta_{i}\left(t\right)\geq \eta_{i}\left(0\right)e^{-(\rho_{3}+c\pi_{i})t}>0.$$

Therefore, $V_{2}\left(t\right)>0$, and noting that $V_{1}\left(t\right)$ is a positive-definite quadratic form, so V(t)>0.

Case 1. During the asynchronous interval $t\in [t_{s},t_{s}+\tau \left(t_{s}\right))$, the controller does not match the communication topology. Compute ${\dot{V}}_{1i}$:

(25) $$\begin{array}{cc} {\dot{V}}_{1i}\left(t\right)= & {\left({\tilde{A}}_{ipq}{\tilde{\varepsilon }}_{i}\left(t\right)+B\tilde{F}\left(x_{i}\left(t\right)\right)\right)}^{T}P_{ip}{\tilde{\varepsilon }}_{i}\left(t\right)+{\tilde{\varepsilon }}_{i}^{T}\left(t\right)P_{ip}\left({\tilde{A}}_{ipq}{\tilde{\varepsilon }}_{i}\left(t\right)+B\tilde{F}\left(x_{i}\left(t\right)\right)\right)\\ \; & -\lambda_{ip}{\tilde{e}}_{i}^{T}K_{q}^{T}B^{T}P_{ip}{\tilde{\varepsilon }}_{i}-\lambda_{ip}{\tilde{\varepsilon }}_{i}^{T}P_{ip}BK_{q}{\tilde{e}}_{i}. \end{array}$$

Apply the Lipschitz condition, $\tilde{F}\left(x\left(t\right)\right)\leq d\tilde{\varepsilon }\left(t\right)$, and it is obtained that

(26) $$\begin{array}{cc} {\dot{V}}_{1i}\left(t\right)\leq & {\tilde{\varepsilon }}_{i}{\left(t\right)}^{T}{\left({\tilde{A}}_{ipq}+d_{N}⊗B\right)}^{T}P_{ip}{\tilde{\varepsilon }}_{i}\left(t\right)+{\tilde{\varepsilon }}_{i}^{T}\left(t\right)P_{ip}\left({\tilde{A}}_{ipq}+d_{N}⊗B\right){\tilde{\varepsilon }}_{i}\left(t\right)\\ \; & -\lambda_{ip}{\tilde{e}}_{i}^{T}K_{q}^{T}B^{T}P_{ip}{\tilde{\varepsilon }}_{i}-\lambda_{ip}{\tilde{\varepsilon }}_{i}^{T}P_{ip}BK_{q}{\tilde{e}}_{i}. \end{array}$$

Compute ${\dot{V}}_{2i}$. According to the normality of orthogonal matrices, $e_{i}^{T}e_{i}={\tilde{e}}_{i}^{T}{\tilde{e}}_{i}$, ${\hat{q}}_{i}^{T}{\hat{q}}_{i}={\tilde{\hat{q}}}_{i}^{T}{\tilde{\hat{q}}}_{i}^{T}$.

(27) $$\begin{array}{cc} {\dot{V}}_{2i} & \geq -\rho_{3}\eta_{i}+c\rho_{1}{\tilde{e}}_{i}^{T}{\tilde{e}}_{i}-c\rho_{2}{\tilde{\hat{q}}}_{i}^{T}{\tilde{\hat{q}}}_{i} \end{array}$$

Combine derivatives:

(28) $$\begin{array}{cc} {\dot{V}}_{i}= & {\dot{V}}_{1i}\left(t\right)+{\dot{V}}_{2i}\left(t\right)\\ \leq & {\tilde{\varepsilon }}_{i}{\left(t\right)}^{T}{\left({\tilde{A}}_{ipq}+d_{N}⊗B\right)}^{T}P_{ip}{\tilde{\varepsilon }}_{i}\left(t\right)+{\tilde{\varepsilon }}_{i}^{T}\left(t\right)P_{ip}\left({\tilde{A}}_{ipq}+d_{N}⊗B\right){\tilde{\varepsilon }}_{i}\left(t\right)\\ \; & -\lambda_{ip}{\tilde{e}}_{i}^{T}K_{q}^{T}B^{T}P_{ip}{\tilde{\varepsilon }}_{i}-\lambda_{ip}{\tilde{\varepsilon }}_{i}^{T}P_{ip}BK_{q}{\tilde{e}}_{i}-\rho_{3}\eta_{i}\left(t\right)+c\rho_{2}{\tilde{\hat{q}}}_{i}^{T}\left(t\right){\tilde{\hat{q}}}_{i}\left(t\right)-c\rho_{1}{\tilde{e}}_{i}^{T}\left(t\right){\tilde{e}}_{i}\left(t\right). \end{array}$$

Compute the Lyapunov bound:

(29) $$\begin{array}{cc} {\dot{V}}_{i}-\beta V_{i}\leq & {\tilde{\varepsilon }}_{i}{\left(t\right)}^{T}{\left({\tilde{A}}_{ipq}+d_{N}⊗B\right)}^{T}P_{ip}{\tilde{\varepsilon }}_{i}\left(t\right)+{\tilde{\varepsilon }}_{i}^{T}\left(t\right)P_{ip}\left({\tilde{A}}_{ipq}+d_{N}⊗B\right){\tilde{\varepsilon }}_{i}\left(t\right)\\ \; & -\lambda_{ip}{\tilde{e}}_{i}^{T}K_{q}^{T}B^{T}P_{ip}{\tilde{\varepsilon }}_{i}-\lambda_{ip}{\tilde{\varepsilon }}_{i}^{T}P_{ip}BK_{q}{\tilde{e}}_{i}-\rho_{3}\eta_{i}\left(t\right)+c\rho_{2}{\tilde{\hat{q}}}_{i}^{T}\left(t\right){\tilde{\hat{q}}}_{i}\left(t\right)\\ \; & -c\rho_{1}{\tilde{e}}_{i}^{T}\left(t\right){\tilde{e}}_{i}\left(t\right)-\beta \eta_{i}\left(t\right)-\beta {\tilde{\varepsilon }}_{i}^{T}P_{ip}{\tilde{\varepsilon }}_{i}. \end{array}$$

Because $\beta >0,\rho_{3}>0$, and ${\tilde{\hat{q}}}_{i}\left(t\right)=-\lambda_{ip}\tilde{\varepsilon }\left(t\right)-\lambda_{ip}\tilde{e}\left(t\right)$, we have

$$\begin{array}{cc} {\dot{V}}_{i}-\beta V_{i}\leq & {\tilde{\varepsilon }}_{i}{\left(t\right)}^{T}{\left({\tilde{A}}_{ipq}+d_{N}⊗B\right)}^{T}P_{ip}{\tilde{\varepsilon }}_{i}\left(t\right)+{\tilde{\varepsilon }}_{i}^{T}\left(t\right)P_{ip}\left({\tilde{A}}_{ipq}+d_{N}⊗B\right){\tilde{\varepsilon }}_{i}\left(t\right)\\ \; & -\lambda_{ip}{\tilde{e}}_{i}^{T}K_{q}^{T}B^{T}P_{ip}{\tilde{\varepsilon }}_{i}-\lambda_{ip}{\tilde{\varepsilon }}_{i}^{T}P_{ip}BK_{q}{\tilde{e}}_{i}+c\rho_{2}{\tilde{\hat{q}}}_{i}^{T}\left(t\right){\tilde{\hat{q}}}_{i}\left(t\right)\\ \; & -c\rho_{1}{\tilde{e}}_{i}^{T}\left(t\right){\tilde{e}}_{i}\left(t\right)-\beta {\tilde{\varepsilon }}_{i}^{T}P_{ip}{\tilde{\varepsilon }}_{i}\\ \leq & {\tilde{\varepsilon }}_{i}{\left(t\right)}^{T}{\left({\tilde{A}}_{ipq}+d_{N}⊗B\right)}^{T}P_{ip}{\tilde{\varepsilon }}_{i}\left(t\right)+{\tilde{\varepsilon }}_{i}^{T}\left(t\right)P_{ip}\left({\tilde{A}}_{ipq}+d_{N}⊗B\right){\tilde{\varepsilon }}_{i}\left(t\right)\\ \; & +{\tilde{e}}_{i}^{T}\left(c\rho_{2}\lambda_{ip}^{2}I-\lambda_{ip}K_{q}^{T}B^{T}P_{ip}\right){\tilde{\varepsilon }}_{i}+{\tilde{\varepsilon }}_{i}^{T}\left(c\rho_{2}\lambda_{ip}^{2}I-\lambda_{ip}P_{ip}BK_{q}\right){\tilde{e}}_{i}\\ \; & -c\rho_{1}{\tilde{e}}_{i}^{T}\left(t\right){\tilde{e}}_{i}\left(t\right)-\beta {\tilde{\varepsilon }}_{i}^{T}P_{ip}{\tilde{\varepsilon }}_{i}. \end{array}$$

Let $\Phi_{i}\left(t\right)={\left[\begin{array}{cc} {\tilde{\varepsilon }}_{i}^{T}\left(T\right) & {\tilde{e}}_{i}^{T}\left(t\right) \end{array}\right]}^{T}$. Due to $\Pi_{i1}<0$, then ${\dot{V}}_{i}-\beta V_{i}\leq \Phi_{i}^{T}\left(t\right)\Pi_{i1}\Phi_{i}\left(t\right)<0$.

Case 2. During synchronous interval $t\in [t_{s}+\tau \left(t_{s}\right),t_{s+1})$, the controller synchronizes the communication topology. Following similar steps,

(30) $$\begin{array}{cc} {\dot{V}}_{i}+\alpha V_{i}\leq & {\tilde{\varepsilon }}_{i}{\left(t\right)}^{T}{\left({\tilde{A}}_{ipp}+d_{N}⊗B\right)}^{T}P_{ip}{\tilde{\varepsilon }}_{i}\left(t\right)+{\tilde{\varepsilon }}_{i}^{T}\left(t\right)P_{ip}\left({\tilde{A}}_{ipp}+d_{N}⊗B\right){\tilde{\varepsilon }}_{i}\left(t\right)\\ \; & -\lambda_{ip}{\tilde{e}}_{i}^{T}K_{p}^{T}B^{T}P_{ip}{\tilde{\varepsilon }}_{i}-\lambda_{ip}{\tilde{\varepsilon }}_{i}^{T}P_{ip}BK_{p}{\tilde{e}}_{i}-\rho_{3}\eta_{i}\left(t\right)+c\rho_{2}{\tilde{\hat{q}}}_{i}^{T}\left(t\right){\tilde{\hat{q}}}_{i}\left(t\right)\\ \; & -c\rho_{1}{\tilde{e}}_{i}^{T}\left(t\right){\tilde{e}}_{i}\left(t\right)+\alpha \eta_{i}\left(t\right)+\alpha {\tilde{\varepsilon }}_{i}^{T}P_{ip}{\tilde{\varepsilon }}_{i}. \end{array}$$

Suppose $\rho_{3}>\alpha >0$, then

(31) $$\begin{array}{cc} {\dot{V}}_{i}+\alpha V_{i}\leq & {\tilde{\varepsilon }}_{i}{\left(t\right)}^{T}{\left({\tilde{A}}_{ipp}+d_{N}⊗B\right)}^{T}P_{ip}{\tilde{\varepsilon }}_{i}\left(t\right)+{\tilde{\varepsilon }}_{i}^{T}\left(t\right)P_{ip}\left({\tilde{A}}_{ipp}+d_{N}⊗B\right){\tilde{\varepsilon }}_{i}\left(t\right)\\ \; & -\lambda_{ip}{\tilde{e}}_{i}^{T}K_{p}^{T}B^{T}P_{ip}{\tilde{\varepsilon }}_{i}-\lambda_{ip}{\tilde{\varepsilon }}_{i}^{T}P_{ip}BK_{p}{\tilde{e}}_{i}+c\rho_{2}{\tilde{\hat{q}}}_{i}^{T}\left(t\right){\tilde{\hat{q}}}_{i}\left(t\right)\\ \; & -c\rho_{1}{\tilde{e}}_{i}^{T}\left(t\right){\tilde{e}}_{i}\left(t\right)+\alpha {\tilde{\varepsilon }}_{i}^{T}P_{ip}{\tilde{\varepsilon }}_{i}. \end{array}$$

And then we obtain

(32) $$\begin{array}{cc} {\dot{V}}_{i}+\alpha V_{i}\leq & {\tilde{\varepsilon }}_{i}{\left(t\right)}^{T}{\left({\tilde{A}}_{ipp}+d_{N}⊗B\right)}^{T}P_{ip}{\tilde{\varepsilon }}_{i}\left(t\right)+{\tilde{\varepsilon }}_{i}^{T}\left(t\right)P_{ip}\left({\tilde{A}}_{ipp}+d_{N}⊗B\right){\tilde{\varepsilon }}_{i}\left(t\right)\\ \; & +{\tilde{e}}_{i}^{T}\left(c\rho_{2}\lambda_{ip}^{2}I-\lambda_{ip}K_{p}^{T}B^{T}P_{ip}\right){\tilde{\varepsilon }}_{i}+{\tilde{\varepsilon }}_{i}^{T}\left(c\rho_{2}\lambda_{ip}^{2}I-\lambda_{ip}P_{ip}BK_{p}\right){\tilde{e}}_{i}\\ \; & -c\rho_{1}{\tilde{e}}_{i}^{T}\left(t\right){\tilde{e}}_{i}\left(t\right)+\alpha {\tilde{\varepsilon }}_{i}^{T}P_{ip}{\tilde{\varepsilon }}_{i}. \end{array}$$

Also, $\Pi_{i2}<0,\rho_{2}\lambda_{ip}^{2}-\rho_{1}<0$, so ${\dot{V}}_{i}+\alpha V_{i}\leq \Phi_{i}^{T}\left(t\right)\Pi_{i2}\Phi_{i}\left(t\right)<0$.

Based on the proof of the synchronous and delayed intervals, it is obtained that

(33) $${\dot{V}}_{i}\leq \left\{\begin{array}{ccc} \beta V_{i}\leq 0, & \; & t\in [t_{s},t_{s}+\tau \left(t_{s}\right))\\ -\alpha V_{i}\leq 0, & \; & t\in [t_{s}+\tau \left(t_{s}\right),t_{s+1}) \end{array}\right.$$

It follows from (3) that V(t) converges to 0 as t→∞. Hence, $\lim_{t\to \infty }{\tilde{\varepsilon }}_{i}\left(t\right)=0,\forall i\in V$, indicating that the nonlinear systems (1) and (2) achieve tracking consensus. □

Based on the above stability analysis, a method for designing stabilizing controller gains is derived. Let $Q=P_{ip}^{-1}$ and Y=KQ,∀p,q∈S. By applying a congruence transformation to $\Pi_{i1}$ and $\Pi_{i2}$ in (1) using $diag\{P_{ip}^{-1},P_{ip}^{-1}\}$, we obtain the following.

$$\Pi_{i3}=\left[\begin{array}{cc} P_{ip}^{-1}{\left({\tilde{A}}_{ipq}+d_{N}⊗B\right)}^{T}+\left({\tilde{A}}_{ipq}+d_{N}⊗B\right)P_{ip}^{-1}+c\rho_{2}\lambda_{ip}^{2}I-\beta P_{ip}^{-1} & *\\ c\rho_{2}\lambda_{ip}^{2}I-\lambda_{ip}P_{ip}^{-1}K_{q}^{T}B^{T} & c(\rho_{2}\lambda_{ip}^{2}-\rho_{1})I \end{array}\right]$$

$$\Pi_{i4}=\left[\begin{array}{cc} P_{ip}^{-1}{\left({\tilde{A}}_{ipp}+d_{N}⊗B\right)}^{T}+\left({\tilde{A}}_{ipp}+d_{N}⊗B\right)P_{ip}^{-1}+c\rho_{2}\lambda_{ip}^{2}I+\alpha P_{ip}^{-1} & *\\ c\rho_{2}\lambda_{ip}^{2}I-\lambda_{ip}P_{ip}^{-1}K_{p}^{T}B^{T} & c(\rho_{2}\lambda_{ip}^{2}-\rho_{1})I \end{array}\right]$$

Bring ${\tilde{A}}_{ipq}=A-\lambda_{ip}BK_{q},{\tilde{A}}_{ipp}=A-\lambda_{ip}BK_{p}$ into

$$\Xi_{i1}=\left[\begin{array}{cc} \Theta_{pq} & *\\ c\rho_{2}\lambda_{ip}^{2}I-\lambda_{ip}Y_{q}^{T}B^{T} & c(\rho_{2}\lambda_{ip}^{2}-\rho_{1})I \end{array}\right]$$

$$\Xi_{i2}=\left[\begin{array}{cc} \Theta_{pp} & *\\ c\rho_{2}\lambda_{ip}^{2}I-\lambda_{ip}Y_{p}^{T}B^{T} & c(\rho_{2}\lambda_{ip}^{2}-\rho_{1})I \end{array}\right]$$

And $\Theta_{pq}=QA^{T}+Q{\left(d_{N}⊗B\right)}^{T}+AQ+\left(d_{N}⊗B\right)Q-\lambda_{ip}\left(BY_{q}+Y_{q}^{T}B^{T}\right)+c\rho_{2}\lambda_{ip}^{2}I-\beta Q$, $\Theta_{pp}=QA^{T}+Q{\left(d_{N}⊗B\right)}^{T}+AQ+\left(d_{N}⊗B\right)Q-\lambda_{ip}\left(BY_{p}+Y_{p}^{T}B^{T}\right)+c\rho_{2}\lambda_{ip}^{2}I+\alpha Q$. From this, we present the following control design scheme.

**Remark** **8.** *An augmented Lyapunov function that incorporates internal dynamic variables from the DETM is constructed in order to eliminate Zeno behavior. Through the regulation effect of this variable, a strictly positive lower bound for event-triggered intervals is guaranteed. To sustain continuous system stability, the function must continue to exhibit its non-increasing property at the moment of communication topology transition.*

**Theorem** **2.** *For given constants $\pi_{i}>0,c>0$, $\rho_{1},\rho_{2}>0$, $\rho_{2}\lambda_{ip}^{2}-\rho_{1}<0$, $\rho_{3}>\alpha >0,\beta >0$, and μ>1, and assuming $c\rho_{2}\lambda_{ip}^{2}I-\lambda_{ip}Y_{q}^{T}B^{T}>0$, suppose there exists a positive-definite matrix Q and matrices $Y_{p}$ such that the inequalities (34) and (35) are satisfied for any (p,q)∈S,p≠q.*

(34) $$\Xi_{i1}<0,\Xi_{i2}<0,$$

(35) $$Q_{ip}<\mu Q_{iq}.$$

*Then, if the switching signal satisfies $h>\frac{\theta T_{m}+ln\mu }{\alpha }$, the system is stable, and the control gain matrix is given by $K_{p}=Y_{p}Q^{-1}$.*

The control gain synthesis procedure is shown in Algorithm 1.
**Algorithm 1:** Asynchronous switching control gain matrix based on DETM**Input:** The matrices $H_{\sigma }$, A,B, related parameters of ADT and DETM**Output:** Controller gain matrices $K_{\sigma }$1**Step 1: Compute transformation matrices $U_{\sigma (t)}$ and $\Lambda_{\sigma (t)}$, then obtain $\lambda_{ip}$**2**Step 2: Validate $\rho_{2}\lambda_{ip}^{2}-\rho_{1}<0$**3**Step 3: Solve LMIs $\Xi_{i1}<0,\Xi_{i2}<0$, then obtain matrices Q and $Y_{p}$**4**Step 4: Ensure $Q_{ip}<\mu Q_{iq}$ and $c\rho_{2}\lambda_{ip}^{2}I-\lambda_{ip}Y_{q}^{T}B^{T}>0$**5**Step 5: Compute control gain matrix $K_{p}=Y_{p}Q^{-1}$**

**Remark** **9.** *We utilize the LMI toolbox to solve the control gain matrices satisfying the conditions of* (34) *and* (35)*, which are designed for both synchronized phases and delayed phases. This method ensures stability under asynchronous switching while lowering design complexity by transforming the Lyapunov stability conditions into LMI constraints and suppressing time-delay disturbances at the same time.*

### 4.3. Feasibility Analysis

To ensure the feasibility of the triggering mechanism, it is necessary to prove that no agent exhibits Zeno behavior. This implies that an infinite number of triggering instances cannot occur within a finite period of time. Therefore, it is necessary to discuss the existence of a positive lower bound for the time between events. In this paper, there are two different types of time sequences: one is the time subsequence induced by the DETM and the other is the time subsequence induced by the switching function.

**Lemma** **4.** *Under a fixed topology, the triggering time intervals generated by the ETM have a positive lower bound.*

**Proof.**  According to the definition of Dini derivatives, for any time instant $t\in [t_{k}^{i},t_{k+1}^{i})$, $∥e_{i}\left(t\right)∥$ is piecewise differentiable. This property implies that

$$\begin{array}{cc} \; & D^{+}∥e_{i}\left(t\right)∥⩽∥{\dot{e}}_{i}\left(t\right)∥=∥{\dot{\hat{x}}}_{i}\left(t\right)-{\dot{x}}_{i}\left(t\right)∥\\ \; & =∥Az_{i}\left(t\right)-A{\hat{x}}_{i}\left(t\right)-Bf\left(x_{i}\left(t\right)\right)-BK\sum_{j=1}^{N}a_{ij}^{\sigma (t)}({\hat{x}}_{j}\left(t\right)-{\hat{x}}_{i}\left(t\right)-BKm_{i\sigma }\left(x_{0}\left(t\right)-{\hat{x}}_{i}\left(t\right)\right)∥\\ \; & ⩽∥A\left(z_{i}\left(t\right)-{\hat{x}}_{i}\left(t\right)\right)+dB\left(z_{i}\left(t\right)-f\left({\hat{x}}_{i}\left(t\right)\right)\right)-BK(\sum_{j=1}^{N}a_{ij}^{\sigma (t)}\left({\hat{x}}_{j}\left(t\right)-{\hat{x}}_{i}\left(t\right)\right)+m_{i\sigma }\left(x_{0}\left(t\right)-{\hat{x}}_{i}\left(t\right)\right))∥\\ \; & ⩽a_{i}∥z_{i}\left(t\right)∥+c_{i}, \end{array}$$

where $a_{i}=∥A∥+d∥B∥$, $c_{i}=∥A{\hat{x}}_{i}\left(t\right)+Bf\left({\hat{x}}_{i}\left(t\right)\right)+BK_{\sigma (t)}{\hat{q}}_{i}∥$.

Further, it is known that $∥e_{i}\left(t\right)∥\leq \frac{c_{i}}{a_{i}}\left(e^{a_{i}\left(t-t_{k}^{i}\right)}-1\right)$. Then, $t-t_{k}^{i}\leq \frac{1}{a_{i}}\ln\left[\frac{a_{i}}{c_{i}}∥e_{i}t)∥+1\right]$.

According to our definition, at the trigger moment, $t_{k}^{i}$, $∥e_{i}\left(t_{k}^{i}\right)∥=0$. The DETM indicates that within the interval $\left[t_{k}^{i},t_{k+1}^{i}\right)$, the inequality $\rho_{3}e_{i}^{T}\left(t\right)e_{i}\left(t\right)-\rho_{2}{\hat{q}}_{i}^{T}\left(t\right){\hat{q}}_{i}\left(t\right)-\pi_{i}\eta_{i}\left(t\right)0$ always holds.

(36) $$\begin{array}{cc} t-t_{k}^{i} & ⩾\frac{1}{a_{i}}\ln\left[\frac{a_{i}}{c_{i}}\sqrt{\pi_{i}\eta_{i}\left(t\right)}+1\right]\\ \; & ⩾\frac{1}{a_{i}}\ln\left[\frac{a_{i}}{c_{i}}\sqrt{\pi_{i}\eta_{i}\left(0\right)e^{-(\gamma_{i}+\theta_{i\phi (t)}\pi_{i})t}}+1\right]. \end{array}$$

The Equation (24) tells us

(37) $$\begin{array}{c} \eta_{i}\left(t_{k+1}^{i}\right)\geq \eta_{i}\left(0\right)e^{-\left(\rho_{3}+c\pi_{i}\right)t_{\infty }^{i}}. \end{array}$$

By combining (36) with (37), the lower bound of the time difference between consecutive triggering instants is derived as $t_{k+1}^{i}-t_{k}^{i}\geq \frac{1}{a_{i}}\ln\left[\frac{a_{i}}{m_{i}\sqrt{∥PB∥}}\sqrt{\pi_{i}\eta_{i}\left(0\right)}e^{-\frac{1}{2}\left(\rho_{3}+c\pi_{i}\right)t_{\infty }^{i}}+1\right]=\phi$. Clearly, ϕ remains strictly positive within any finite time interval. □

We first consider the time subsequence generated by the DETM.

Case 1. During the interval $\left[t_{k}^{i},t_{k+1}^{i}\right)$, the communication topology remains unchanged. According to Lemma 4, there is no Zeno behavior within this interval.

Case 2. If the topology structure undergoes *n* changes during the interval $\left[t_{k}^{i},t_{k+1}^{i}\right)$, let $t_{k,\sigma }^{i}$ denote the triggering instants caused by topology switching, forming a switching time sequence $t_{k}^{i}<t_{k,\sigma }^{i}<t_{k,\sigma +1}^{i}<\ldots <t_{k,\sigma +n}^{i}$. The time intervals are divided into three scenarios. First, consider the subinterval $\left[t_{k}^{i},t_{k,\sigma }^{i}\right)$. Although this is an open interval, neither event triggering nor topology switching occurs within it, thereby imposing constraints on the number of triggering occurrences during this period.

In the second scenario, within the subinterval $\left[t_{k,\sigma }^{i},t_{k,\sigma +n}^{i}\right)$, no event triggering occurs and the topology structure undergoes only a finite number of switches. Since this does not satisfy the definition of Zeno behavior, the possibility of a Zeno phenomenon is ruled out.

For the third scenario, the communication topology remains unchanged over the interval $\left[t_{k,\sigma +n}^{i},t_{k+1}^{i}\right)$. In this case, the system evolution is equivalent to the first scenario, allowing the same proof method to exclude Zeno behavior.

In summary, the triggering time sequence generated by the DETM is free from Zeno phenomena.

Then, consider the time subsequence generated by the ADT. According to Definition 1, the number of topology switches is finite within any finite time interval. Therefore, Zeno phenomena cannot occur because the time subsequence caused by topology switching does not possess the attribute of infinite accumulation.

Furthermore, existing studies have established that if multiple time sequences individually do not exhibit Zeno behavior, their combination will also not induce Zeno phenomena [29]. Based on the preceding analysis, the time sequences generated by the DETM and the switching functions have been proven to be free from Zeno behavior. Consequently, we can assert that the entire system avoids Zeno phenomena, meaning no agent will experience infinite triggering within any finite time interval. This completes the proof.

## 5. Simulation

### 5.1. Example 1

This section illustrates the validity of the proposed distributed control strategy through a numerical simulation. Consider a nonlinear MAS has one leader and five followers, labeled as agents 0–5, where agent 0 serves as the leader. The system matrices satisfying assumption (1) are given as follows:

$$A=\left(\begin{array}{ccc} -0.2 & 1 & 0\\ -1 & 0 & -1\\ 0 & 0 & -1 \end{array}\right),B=\left(\begin{array}{c} 1\\ 1\\ 1 \end{array}\right).$$

**Figure 4:**
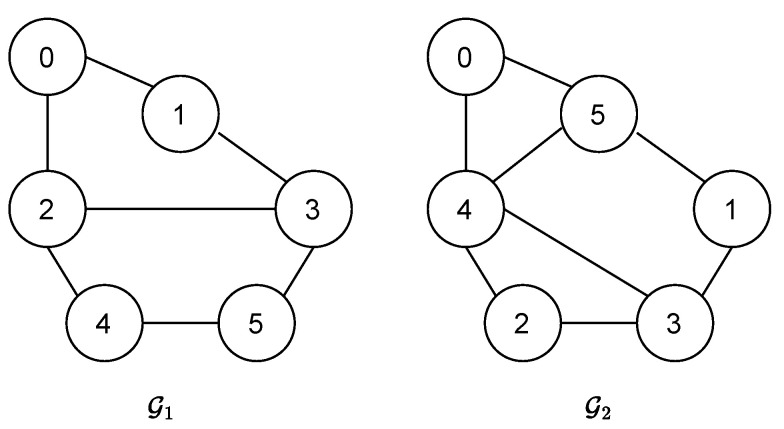
Switching topologies.

The control process involves two possible topologies, $G_{1}$ and $G_{2}$, governed by the switching signal σ(t)∈{1,2}, as shown in Figure 4.

From Figure 4, the Laplacian matrix $L_{\sigma (t)}$ and the degree matrix $D_{\sigma (t)}$ can be obtained, based on which matrix $H_{\sigma (t)}$ is computed as follows.

$$H_{1}=\left(\begin{array}{ccccc} 3 & 0 & -1 & -1 & 0\\ 0 & 3 & -1 & -1 & 0\\ -1 & -1 & 2 & 0 & 0\\ -1 & -1 & 0 & 3 & -1\\ 0 & 0 & 0 & -1 & 1 \end{array}\right),H_{2}=\left(\begin{array}{ccccc} 2 & 0 & -1 & 0 & -1\\ 0 & 3 & -1 & -1 & -1\\ -1 & -1 & 3 & -1 & 0\\ 0 & -1 & -1 & 4 & -1\\ -1 & -1 & 0 & -1 & 4 \end{array}\right).$$

Based on the matrices, the corresponding transformation matrices $U_{\sigma (t)}$ and $\Lambda_{\sigma (t)}$ in Equation (11) are computed as follows.

$$U_{1}=\left(\begin{array}{ccccc} -0.31 & -0.32 & -0.24 & -0.71 & 0.5\\ -0.31 & -0.32 & -0.24 & 0.71 & 0.5\\ -0.36 & -0.63 & 0.59 & 0 & -0.34\\ -0.48 & 0 & 0.64 & 0 & 0.6\\ -0.67 & 0.64 & 0.35 & 0 & -0.15 \end{array}\right),\;U_{2}=\left(\begin{array}{ccccc} -0.53 & 0.79 & 0.1 & -0.13 & 0.3\\ -0.5 & -0. & -0.18 & -0.67 & 0.2\\ -0.5 & -0.07 & -0.76 & 0.09 & -0.4\\ -0.36 & -0.33 & 0.06 & 0.68 & 0.54\\ -0.37 & -0.03 & 0.62 & 0.25 & -0.66 \end{array}\right).$$

Subsequently, under the same parameter settings as in (19), with μ=1.2 and $T_{m}=0.5$, various combinations of α and β are used to obtain the corresponding minimum ADT *h*. The results are, respectively, presented in Table 1 and Table 2.

Table 1 shows that the minimum ADT $\tau_{\alpha }$ depends on α, where smaller α values lead to larger $\tau_{\alpha }$. As shown in Table 2, for a fixed α, higher values of β result in an increase in $\tau_{\alpha }$. Therefore, the ADT parameters are selected according to this relationship in Table 3, while the DETM parameters are derived from $\Lambda_{1}$, $\Lambda_{2}$, and Theorem 2. Accordingly, the resulting feedback gains are $K_{1}=\left[0.417,0.147,0.1966\right]$ and $K_{2}=\left[0.649,0.217,0.31\right]$. The initial values of the internal dynamic variables are set as $\eta_{i}\left(0\right)=\left(0.4,0.2,0.5,0.3,0.6\right)$.

According to the analysis in (2), the tracking consensus of the agents in Figure 4 can be transformed into the asymptotic consensus of the tracking error in system (10). The initial value of the transformed tracking error, obtained via the orthogonal matrix $U_{\sigma (t)}$, is set to ${\tilde{\varepsilon }}_{1}=\left(3,-5,-8\right)$, ${\tilde{\varepsilon }}_{2}=\left(-3,5,-2\right)$, ${\tilde{\varepsilon }}_{3}=\left(6,-10,3\right)$, ${\tilde{\varepsilon }}_{4}=\left(1,6,5\right)$, and ${\tilde{\varepsilon }}_{5}=\left(-6,4,4\right)$. The nonlinear function is denoted as $f\left({\tilde{\varepsilon }}_{i}\left(t\right),t\right)=0.1(|{\tilde{\varepsilon }}_{i1}\left(t\right)+{\tilde{\varepsilon }}_{i2}\left(t\right)+{\tilde{\varepsilon }}_{i3}\left(t\right)-1|-|{\tilde{\varepsilon }}_{i1}\left(t\right)+{\tilde{\varepsilon }}_{i2}\left(t\right)+{\tilde{\varepsilon }}_{i3}\left(t\right)+1\left|\right)$.

**Figure 5:**
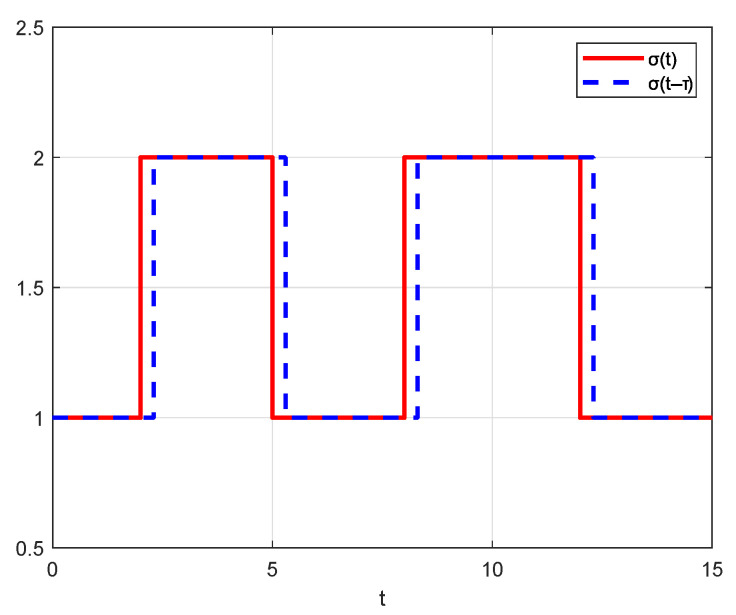
Asynchronous switching signal in Example 1.

Figure 5, respectively, illustrates the switching signals of the communication topology and the controller, σ(t) and σ(t−τ).

**Figure 6:**
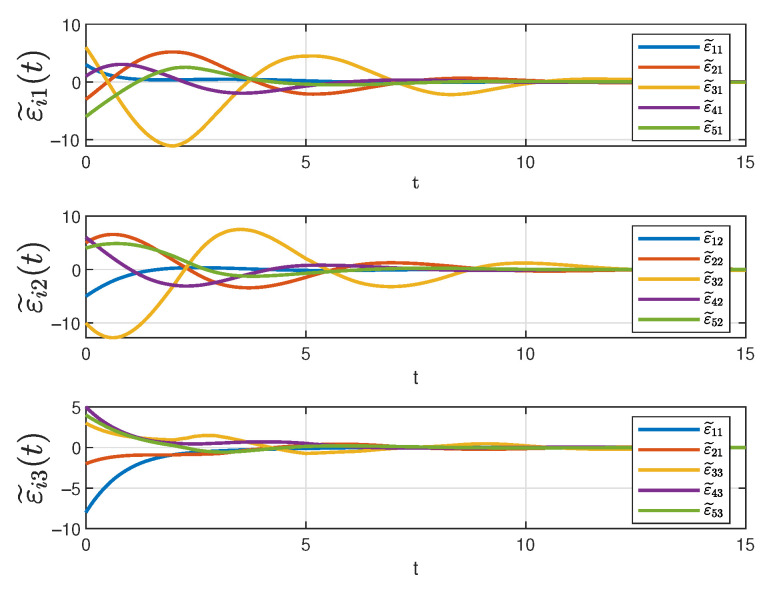
The status of system (10).

Figure 6 depicts the evolution of the three state components in system (10) over time under the designed controller. In the figure, solid lines of different colors represent different state trajectories. It can be seen that all the trajectories converge at 12s even though the initial states are different. As previously analyzed, the different trajectories correspond to the tracking errors, after orthogonal transformation, of different followers with respect to the same leader. Therefore, it can be concluded that all the tracking errors gradually decrease over time and eventually converge to zero, ensuring that the desired tracking objectives for each state component are achieved in the controller (14).

**Figure 7:**
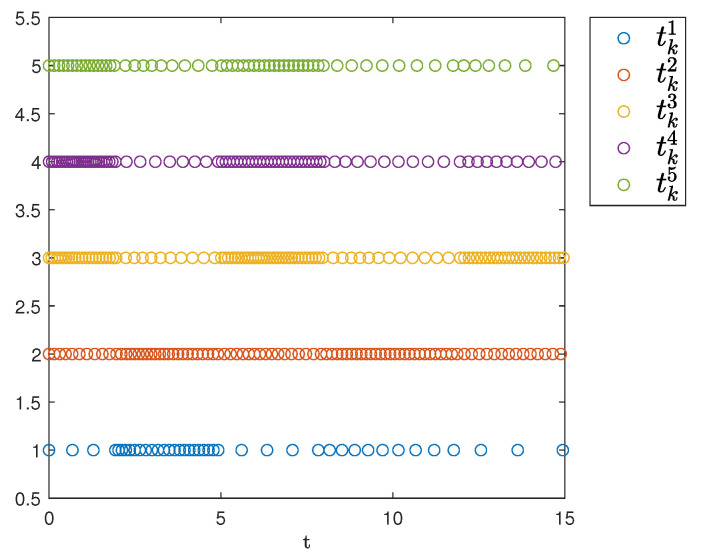
Event-triggering times.

Figure 7 shows the triggering instants of the followers. As illustrated, the figure clearly displays the time intervals for each agent and indicates that Zeno behavior does not occur.

**Figure 8:**
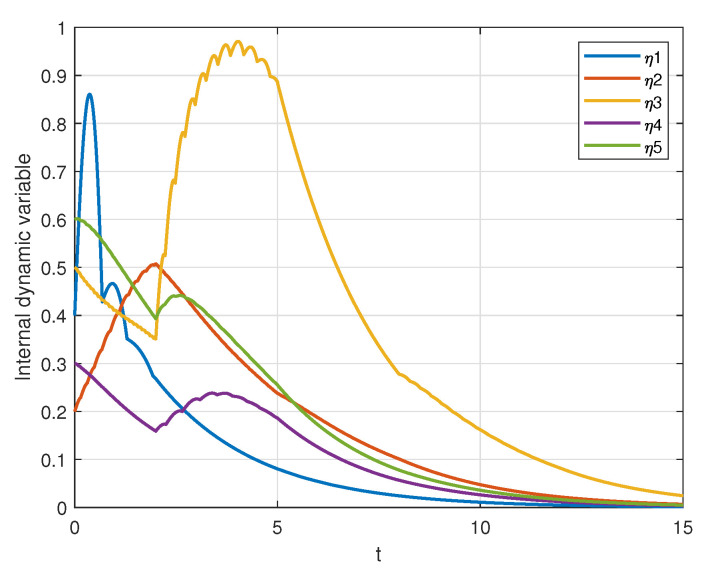
Evolutions of the internal dynamic state.

In addition, under the triggering condition (8), the evolution curves of the internal dynamic variables are shown in Figure 8. As indicated in the figure, the internal dynamic variables gradually approach zero over time.

**Figure 9:**
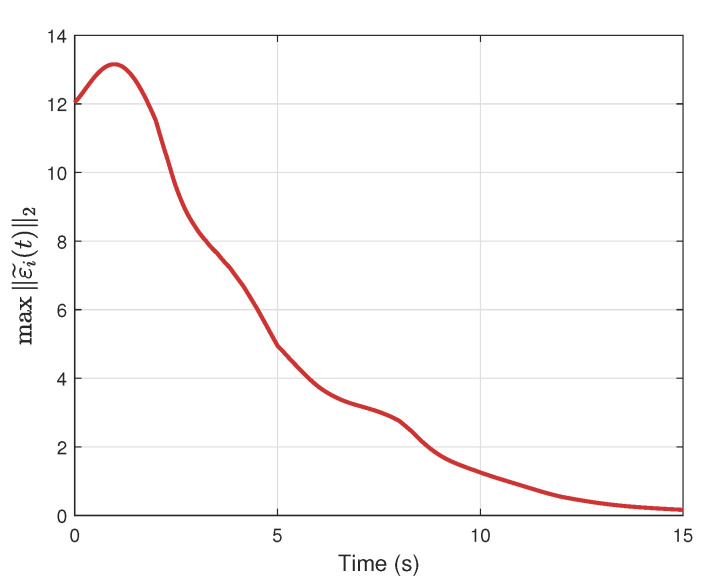
Maximum state norm over agents.

Figure 9 presents the evolution of the maximum tracking error among the agents, which converges asymptotically to zero over time.

**Figure 10:**
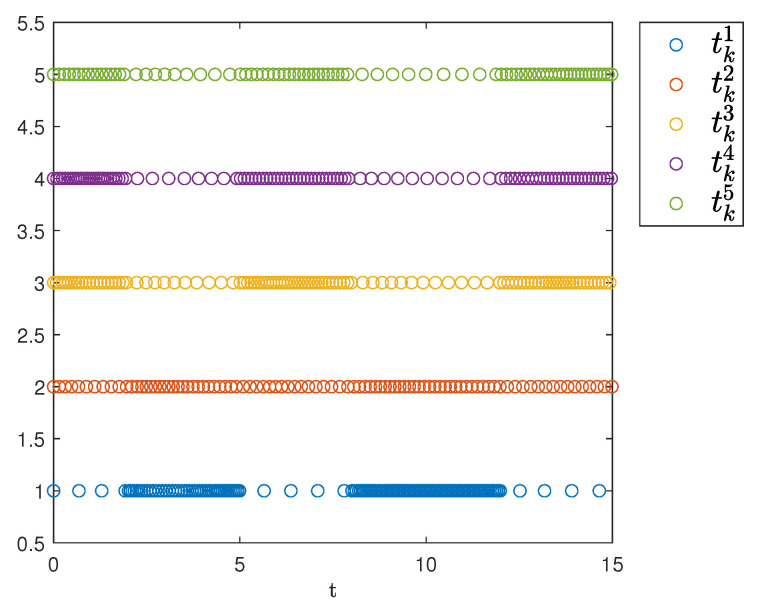
Triggering times of static event-triggered mechanism.

For comparison, a simulation was conducted using a SETM. In this design, setting $\pi_{i}=0$ indicates the exclusion of internal dynamic variables. The triggering times under the SETM are shown in Figure 10. As observed in the figure, the introduction of the internal dynamic variable $\pi_{i}$ significantly reduces the number of trigger events.

To compare the number of triggering events between the two mechanisms, Table 4 records the triggering times of the agents using the SETM and DETM. As shown in Table 4, the DETM is more efficient than the SETM in terms of energy-saving.

### 5.2. Example 2

In this example, three extensions are implemented based on the simulation of Example 1. First, the communication topology switching signal is generated by a Markov chain, replacing the original piecewise constant function with a stochastic switching pattern. Second, the fixed delay between the topology and controller switching signals is transformed into a time-varying delay τ(t)=0.1+0.2sin(t). Third, the nonlinear function is set as $f\left(\tilde{\varepsilon }\left(t\right),t\right)=0.1\cos\left(\tilde{\varepsilon }+\sin\left(t\right)\right)$. Here, the communication topologies $G_{1}$ and $G_{2}$ and all the initial parameters are the same as in Example 1.

**Figure 11:**
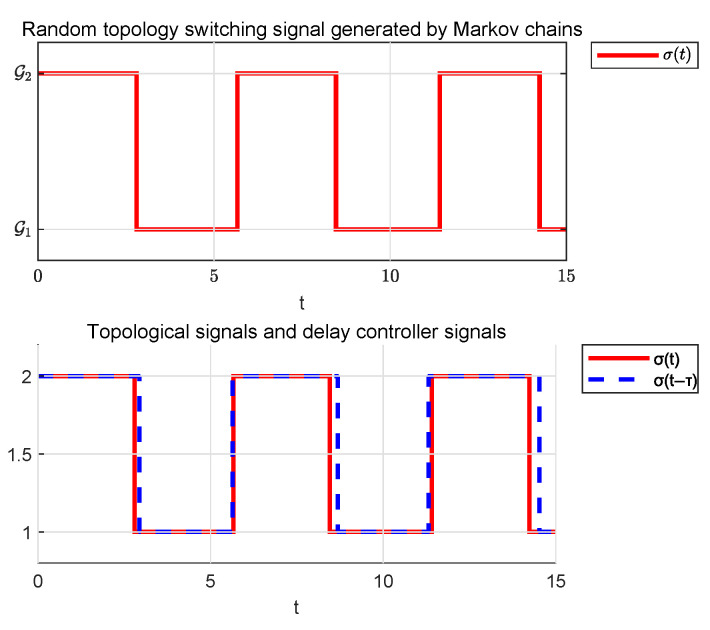
Asynchronous switching signal 1.

**Figure 12:**
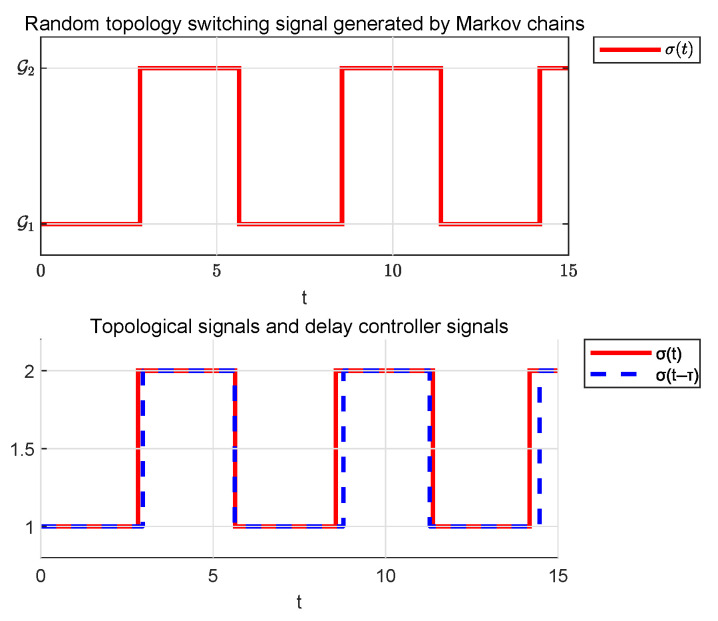
Asynchronous switching signal 2.

The Markov transfer probability matrix is set as $P=\left(\begin{array}{cc} 0.85 & 0.15\\ 0.25 & 0.75 \end{array}\right)$. Figure 11 and Figure 12 demonstrate two stochastic communication topology switching signals generated by a Markov chain. Their corresponding state trajectories and event-triggering sequences will be presented separately in subsequent figures.

**Figure 13:**
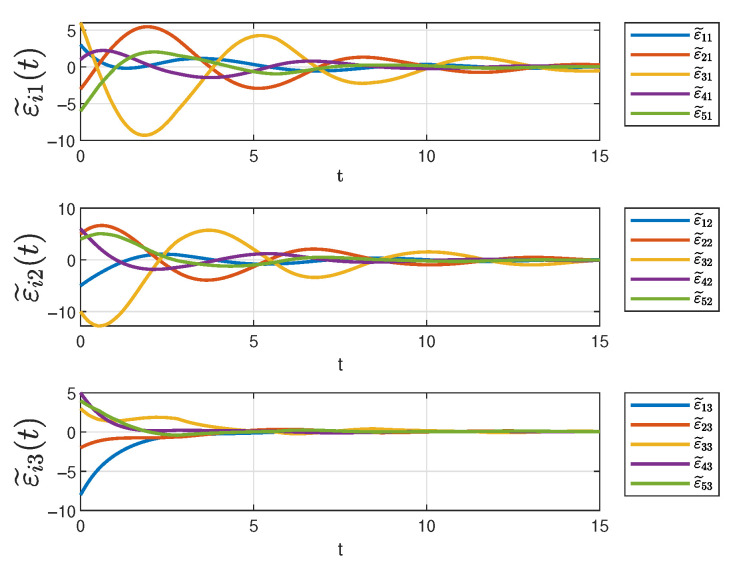
The state trajectories 1.

**Figure 14:**
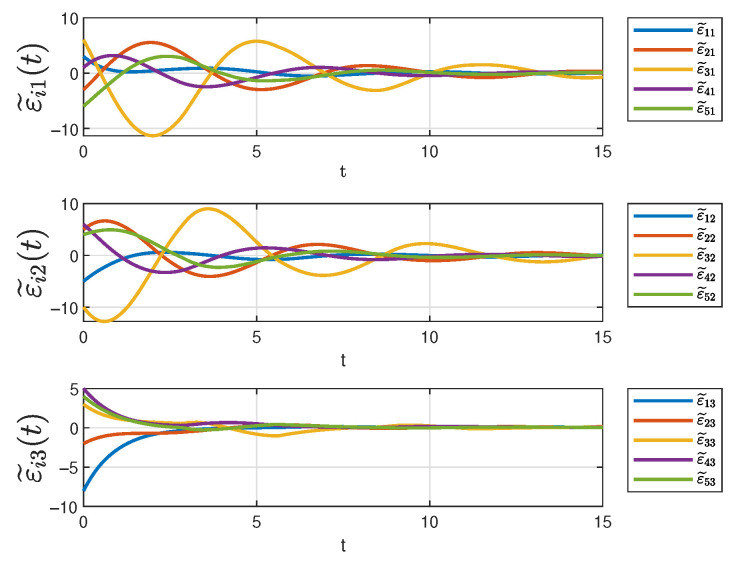
The state trajectories 2.

Figure 13 and Figure 14 depict the evolution of three state components in system (10) under different switching signals. The figures demonstrate that all trajectories eventually converge despite differing initial states.

**Figure 15:**
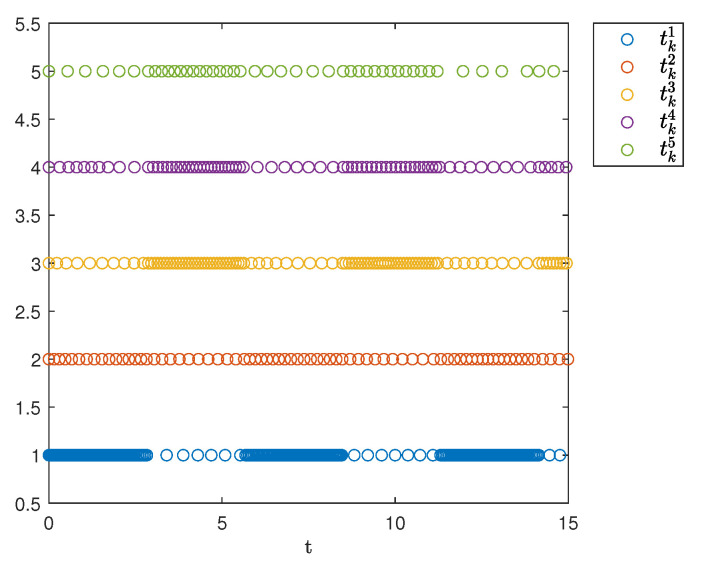
Event-triggered sequences 1.

**Figure 16:**
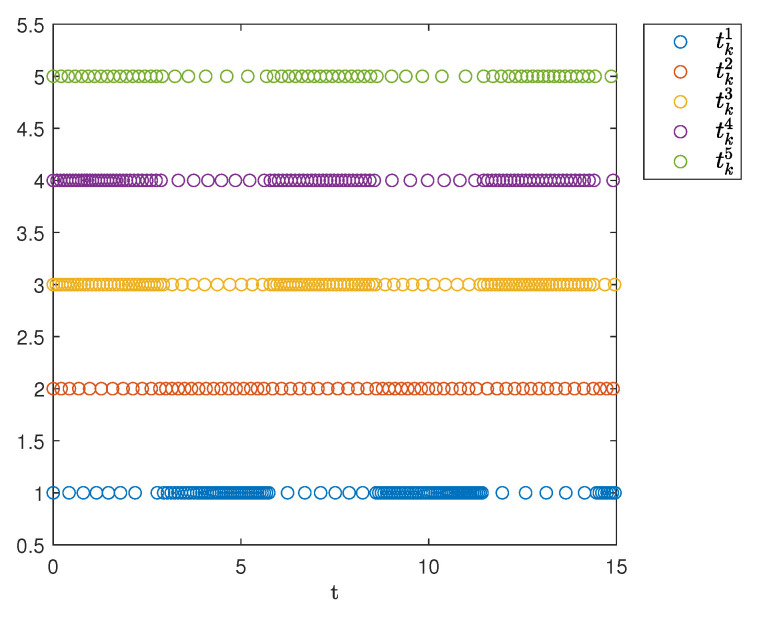
Event-triggered sequences 2.

Figure 15 and Figure 16 display the temporal distribution of triggering instants for the followers, illustrating the event-triggered control throughout the simulation period. Each trigger event is discretely labeled on the time axis, and its sparse distribution proves that the number of controller updates has been reduced.

To compare the number of triggering events between the signals, Table 5 records the triggering numbers of the agents. As shown in Table 5, the topology switching signal 1 yields more triggers.

## 6. Conclusions

This study investigates the tracking consensus of nonlinear MASs under asynchronous switching. Adopting a leader–follower framework, the ADT method is extended to asynchronous switching systems. To address resource consumption caused by frequent controller updates, an event-triggered control strategy is designed to achieve optimization of communication and control. However, current research is constrained by the requirement that system nonlinearities must satisfy Lipschitz conditions, limiting its applicability to nonlinear systems. Additionally, the topology switching involves only finite modes and is not sufficiently adaptable to dynamic topologies with continuous unknown changes. Future work will focus on overcoming non-Lipschitz constraints by developing adaptive triggering mechanisms and exploring dynamic topology prediction methods based on reinforcement learning, thereby offering new solutions for cooperative control in complex environments. 

## Figures and Tables

**Table 1:** Minimum average dwell time *h* corresponding to different values of α (β=1).

α	0.1	0.5	1.0
*h*	7.32	1.86	1.18

**Table 2:** Minimum average dwell time *h* corresponding to different values of β (α=0.3).

β	0.1	0.5	1.0
*h*	0.27	0.94	2.77

**Table 3:** The parameter values in the ADT method and DETM.

μ	$T_{m}$	α	β	*c*	$\rho_{1}$	$\rho_{2}$	$\rho_{3}$	$\pi_{i}$
1.2	0.5	0.3	1	0.1	1	0.01	0.4	0.01

**Table 4:** Triggering numbers of the agent in Example 1.

	$\pi_{i}$	1	2	3	4	5
SETM	0	139	93	109	106	84
DETM	0.01	36	53	64	59	34
Percentage reduction		74.1%	43.0%	41.3%	44.3%	59.5%

**Table 5:** Triggering numbers of the agent in Example 2.

	Total Numbers	1	2	3	4	5
Topology switching signal 1	524	223	73	103	77	48
Topology switching signal 2	472	118	69	118	102	65

## Data Availability

Data are contained within the article.
